# Adaptive Müller cell responses to microglial activation mediate neuroprotection and coordinate inflammation in the retina

**DOI:** 10.1186/1742-2094-8-173

**Published:** 2011-12-07

**Authors:** Minhua Wang, Wenxin Ma, Lian Zhao, Robert N Fariss, Wai T Wong

**Affiliations:** 1 Unit on Neuron-Glia Interactions in Retinal Diseases, Office of the Scientific Director, National Eye Institute, National Institutes of Health, Bethesda, MD, USA; 2 Biological Imaging Core, National Eye Institute, National Institutes of Health, Bethesda, MD, USA

**Keywords:** Müller cell, microglia, retina, cytokine, cellular interaction, gliosis, migration, adhesion, inflammation, neuroprotection

## Abstract

**Purpose:**

Microglia and Müller cells are prominent participants in retinal responses to injury and disease that shape eventual tissue adaptation or damage. This investigation examined how microglia and Müller cells interact with each other following initial microglial activation.

**Methods:**

Mouse Müller cells were cultured alone, or co-cultured with activated or unactivated retinal microglia, and their morphological, molecular, and functional responses were evaluated. Müller cell-feedback signaling to microglia was studied using Müller cell-conditioned media. Corroborative *in vivo *analyses of retinal microglia-Müller cell interactions in the mouse retina were also performed.

**Results:**

Our results demonstrate that Müller cells exposed to activated microglia, relative to those cultured alone or with unactivated microglia, exhibit marked alterations in cell morphology and gene expression that differed from those seen in chronic gliosis. These Müller cells demonstrated *in vitro *(1) an upregulation of growth factors such as GDNF and LIF, and provide neuroprotection to photoreceptor cells, (2) increased pro-inflammatory factor production, which in turn increased microglial activation in a positive feedback loop, and (3) upregulated chemokine and adhesion protein expression, which allowed Müller cells to attract and adhere to microglia. *In vivo *activation of microglia by intravitreal injection of lipopolysaccharide (LPS) also induced increased Müller cell-microglia adhesion, indicating that activated microglia may translocate intraretinally in a radial direction using Müller cell processes as an adhesive scaffold.

**Conclusion:**

Our findings demonstrate that activated microglia are able to influence Müller cells directly, and initiate a program of bidirectional microglia-Müller cell signaling that can mediate adaptive responses within the retina following injury. In the acute aftermath following initial microglia activation, Müller cell responses may serve to augment initial inflammatory responses across retinal lamina and to guide the intraretinal mobilization of migratory microglia using chemotactic cues and adhesive cell contacts. Understanding adaptive microglia-Müller cell interactions in injury responses can help discover therapeutic cellular targets for intervention in retinal disease.

## Background

The response of the central nervous system (CNS) to disease, inflammation, and injury features prominent involvement of astrocytes, the "macroglia" population of the CNS [1], as well as microglia, the primary resident immune cell population [2]. While the astrocytic and microglial responses in these pathogenic contexts have been thought to involve cross-talk between these two cell populations [3], the mechanisms and functional significances underlying these interactions are incompletely understood [4,5]. In the retina, Müller cells, the radial astroglia of the retina, as well as retinal microglia, similarly demonstrate marked changes in various retinal injuries and diseases [6,7]. Müller cells and microglial injury responses in the retina have been ascribed both beneficial and deleterious features [8-10], and the "Janus-faced" nature of these responses may be secondary to the complex system of communications between microglia and macroglia. How these two cells types interact in the aftermath of retinal injury, and how they shape an adaptive or maladaptive overall response have not been fully explored.

In the uninjured state, microglia in the brain [11,12] and the retina [13] demonstrate dynamic motility in their processes that are likely to subserve immune surveillance [14]. Time-lapse imaging in live tissue has also shown that in the minutes following tissue injury, microglia in the vicinity of injury polarize their processes to the injury site and initiate positive chemotaxis and aggregation [11-13]. Based on these and other observations [15-17], the activation of microglia in the acute aftermath of CNS injury is generally thought to constitute the initial step of a generalized inflammatory response, and precede responses in astroglia. An understanding of how the overall injury response, initiated following microglial activation, is subsequently shaped by communications between these two cell types is likely central to elucidating the functional importance of post-injury inflammation and the mechanisms underlying chronic neuroinflammatory changes thought to drive CNS pathologies, including retinal disease [10].

In the current study, we aimed to examine the existence, nature, and functional significance of microglia- Müller cell interactions following microglial activation. We employed an *in vitro *co-culture system where morphological, molecular, and functional alterations in cultured Müller cells were examined following juxtaposition with activated and non-activated microglial cells. We also sought corroboration for *in vitro *observations with *in vivo *examinations of microglia- Müller cell interactions in the retina following microglial activation. Our observations demonstrated that Müller cells exhibited prominent molecular and functional responses to microglial activation that appeared to be adaptive in nature and which differed from the changes found in chronic gliosis. We also found evidence for bidirectional signaling between microglia and Müller cells that facilitated further activation, migration, and cell adhesion of microglia that may be relevant to amplifying and coordinating an inflammatory response in the retina. These data underline a program of injury response in the retina constituted by microglia- Müller cell interactions that likely serve to provide protection to surrounding neurons and to restore tissue homeostasis.

## Methods

### Culture of mouse retinal Müller cells

Experiments were conducted according to protocols approved by the NEI Institutional Animal Care and Use Committee and adhered to the ARVO Statement for the Use of Animals in Ophthalmic and Vision Research. Experimental animals were housed and bred in National Institutes of Health animal facilities.

Retinal cell cultures were obtained using the retinas of wild type C57BL/6 mice (Charles River Laboratories Inc., Wilmington, MA). Müller cells were isolated from the retinas of postnatal day (P)7-12 C57BL/6 mice using a protocol modified from Hicks and Courtois [18]. Following euthanasia, mice were rapidly enucleated and their globes immersed in Dulbecco's modified Eagle's medium (DMEM) containing 1:1000 penicillin/streptomycin overnight in the dark at room temperature. These were subsequently transferred into 0.1% trypsin at room temperature for 60 minutes and then rinsed thrice with DMEM containing 10% fetal bovine serum (FBS, Gibco/Invitrogen, Carlsbad, CA) to terminate the digestion reaction. The retinas were carefully dissected free from the other ocular tissues and dissociated by trituration. The resulting cell suspension was then seeded into 75 cm^2 ^flasks (4-5 retinas per flask) containing DMEM medium with 10% FBS at 37°C. The culture medium (DMEM medium with 10% FBS) was changed 24 hours after seeding. At 3-4 days intervals, cultures were shaken vigorously to detach non-adherent cells which were then removed from the culture by aspiration. When the remaining adherent cells in the cultures reached 80% confluence, they were detached from the flask bottoms using 0.1% trypsin, resuspended in fresh DMEM containing 10% FBS, and replated into new flasks. Immunohistochemical staining of these remaining cells demonstrated them to be composed of > 98% Müller cells as evidenced by immunopositivity for glutamine synthetase (GS), glutamate aspartate transporter (GLAST), vimentin, as well as immunonegativity for CD11b (microglia), NeuN (neurons), and GFAP (astrocytes).

### Co-culture of retinal microglia and Müller cells

Mouse retinal microglia cells were isolated from retina of young C57BL/6 mice (P10-30) and evaluated for purity as previously described [19]. Cultured microglia were collected and seeded at a density of 0.5-1 × 10^6^/well in 10% FBS DMEM onto Transwell permeable support membrane inserts (Corning, Corning, NY) and allowed to settle and grow for the next 24 hours. These cell-bearing inserts constituted the upper chamber in a two-chambered microglia-Müller cell co-culture system. Cultured Müller cells were seeded into the bottom of 6-well plates at a density of 0.5-1 × 10^5 ^cells/well in 10% FBS DMEM. After 24 hours, Müller cells were washed with DMEM and replaced with 0.5 ml of DMEM containing 5% heat-inactivated (HI) serum. These Müller cells were then co-cultured with microglia-containing inserts for 48 hours in the following configurations: (1) Müller cells incubated with empty inserts lacking microglia (control), (2) Müller cells incubated with inserts containing microglia that had not been exposed to LPS, and (3) Müller cells incubated with inserts containing activated microglia which had been previously exposed to 1 μg/ml of lipopolysaccharide (LPS) (1 μg/ml, Sigma, St Louis, MO) for 6 hrs and then rinsed thoroughly three times with DMEM to remove all residual LPS before co-culture.

Following co-culture, the two co-culture chambers were disassembled and exposed Müller cells were either: (1) washed with PBS and harvested for mRNA analysis, (2) fixed in 4% paraformaldehyde in PBS for 60 minutes for subsequent immunohistochemical and labeling studies, or (3) washed thrice with DMEM and incubated in fresh DMEM containing 5% HI-serum for an additional 24 hours to generate conditioned media.

### Immunohistochemistry

Immunohistochemistry was performed on cultured Müller and microglial cells as well as mouse retinal sections. Cultured cells were fixed in 4% paraformaldehyde in PBS at room temperature for 30-60 minutes and washed thrice with PBS. Retinal sections were prepared from fixed eyecups of experimental animals which had been cryoprotected in 30% sucrose in PBS for 24 hrs, embedded in OCT (Tissue-Tek, Elkhart, IN), and flash frozen in acetone containing dry ice. Retinal sections of 30- μm thickness were cut on a cryostat (Leica CM 3050S, Buffalo Grove, IL) and stored at -20°C. Sections were thawed and rinsed with PBS prior to immunohistochemical staining.

Cultured cells or retinal sections were pre-incubated in blocking buffer (consisting of 10% normal goat serum (NGS), 5% bovine serum, and 0.5% Triton X-100 in 1 × PBS) for 2 hours at room temperature, and then in primary antibody (diluted in the blocking buffer) overnight at room temperature. Primary antibodies targeting the following molecules were used: glutamine synthetase (GS, clone: GS-6), 1:200; NeuN (clone: A60), 1:200; glutamate aspartate transporter (GLAST), 1:200 (all from Chemicon International, Temecula, CA); glial fibrillary acidic protein (GFAP, clone: 2.2b10), 1:200 for cell culture, 1:800 for retinal sections; CD11b (clone: 5C6), 1:200; F4/80 (clone: A3-1), 1:200 (all from AbD Serotec, Raleigh, NC); ionized calcium binding adaptor molecule-1 (Iba1), 1:800 (Wako, Richmond, VA); vimentin, 1:200 to 1:800 (clone: V9, MP Biomedicals, Solon, OH and AbCam, Cambridge, MA). The cells or sections were then washed 3 times with 1 × PBS, and incubated in secondary antibody (1:400) for 1 hr at room temperature. The following secondary antibodies were used: Alexa 488-conjugated goat anti-rabbit IgG antibody, Alexa 488-conjugated goat anti-chicken IgG antibody, Cy3- conjugated goat anti-mouse IgG antibody, Cy5- conjugated goat anti-mouse IgG, Cy3- conjugated goat anti-rat IgG and Cy3- conjugated goat anti-guinea pig IgG antibody (all from Invitrogen, Carlsbad, CA). F-actin in cultured cells was labeled using AlexaFluor 555-conjugated Phalloidin (1:100, from Invitrogen, Carlsbad, CA) by incubating for 2 hours at room temperature. After immunolabelling, cells or sections were rinsed thrice in 1 × PBS and coverslipped in Vectashield mounting medium containing 4',6-diamidino-2-phenylindole (DAPI) (Vector Lab Inc, Burlingame, CA).

### Confocal microscopy and image analysis of microglia and Müller cells in retinal sections

Retinal sections following immunohistochemical staining were imaged with confocal microscopy (FluoView 1000, Olympus, Japan). Multiplane z-series were collected using a 40× or 63×, oil-immersion objective. Each z-series spanned 30 μm in depth, and comprised of 30-50 images per series, each spaced 0.6-1 μm apart. Image stacks were analyzed with an image analysis software package (Volocity, Perkin Elmer, Waltham, MA) to generate a 3-dimensional rendering of the *in vivo *spatial relationships between microglia and Müller cells.

### Morphological analysis of cultured Müller cells

Cultured Müller cells following co-culture were immunolabeled for glutamine synthetase, phallodin and DAPI, and imaged with an epifluorescence microscope (Olympus BX51, Center Valley, PA). Computer-based analysis of the morphology of individual cells was performed with NIH ImageJ (Bethesda, MD, Version 1.441) using the "Analyze Particle" function. Morphological parameters included: cellular area, perimeter, circularity (defined as 4π(area of cell)/(cell perimeter^2^)), and elongation factor (defined as the ratio of the major axis to the minor axis of the cell).

### Semi-quantitative rt-PCR

Lysates of cultured cells were homogenized by using QIAshredder spin column (Qiagen, Valencia, CA) and total RNA isolated using the RNeasy Mini kit (Qiagen) according to the manufacturer's specifications. Isolated RNA was treated with RNase-free DNase I (Qiagen) to remove any contaminating genomic DNA. First-strand cDNA synthesis from mRNA was performed using a cDNA synthesis kit (Ambion, Austin, TX) using oligo-dT as the primer. RT-PCR was performed using a 15 μl reaction cocktail in conjunction with 1 μl of the cDNA, 2 μl of primer mixture, and 12 μl of HotStarTaq Plus DNA Polymerase (Qiagen). After an initial denaturation at 95°C for 5 minutes, PCR amplification was conducted for 15-40 cycles. The cycle numbers for quantification of each product were chosen within the linear range of the PCR. GAPDH was used as an internal control. The stability of levels of GAPDH mRNA across experimental conditions was verified by comparison to two other house-keeping genes, β-actin and 18S rRNA. The absence of contaminating genomic DNA in the isolated RNA following DNase treatment was confirmed by performing the PCR amplification in the absence of reverse transcriptase in parallel control reactions and by ascertaining that no amplification product was formed. The primer sequences for the PCR reactions are provided in Table 1.

**Table 1:** DNA primers used in rt-PCR amplification reactions

	Forward (5'-3')	Reverse (5'-3')
bFGF	atggctgccagcggcatcac	tcagctcttagcagacattgga

BDNF	ggactctggagagcgtgaa	ggtcagtgtacatacacagg

CX3CL1	ttcacgttcggtctggtggg	ggttcctagtggagctaggg

CCL2	gcaggtccctgtcatgctt	ctagttcactgtcacactgg

CCL3	ccaagtcttctcagcgccat	ggttgaggaacgtgtcctg

CNTF	ggctttcgcagagcaatcac	gcttggccccataatggct

GAPDH	cctctggaaagctgtggcg	gttgctgtagccgtattcatt

GDNF	atgggattcgggccacttgg	tcagatacatccacaccgtttag

GFAP	ttcctgtacagactttctcc	cccttcaggactgccttagt

Glast	ctctgggcatcctcttcttg	caaatctggtgatgcgtttg

GS	tgcctgcccagtgggaatt	tattggaagggttcgtcgcc

ICAM-1	ccaattcacactgaatgccag	ggcttgtcccttgagttttatg

IFN-γ	ctggtggttgctcctcttac	ctcctgggcctctcctgtg

IL-1β	gccaccttttgacagtgatgag	ttaggaagacacagattccatg

IL-6	atgaagttcctctctgcaagag	ctaggtttgccgagtagatctc

iNOS	cctcccagccttgcatcc	cagagcctcgtggctttgg

LIF	aatgccacctgtgccatacg	caacttggtcttctctgtcccg

NGF	ggcgtacaggcagaaccgta	cagcctcttcttgtagccttc

TGF-β	ccactgatacgcctgagtg	gctgcacttgcaggagcg

TNF-α	atgagcacagaaagcatgatc	tcacagagcaatgactccaaag

VCAM-1	ggataccagctcccaaaatcc	cactttggatttctgtgcctc

Vimentin	gtacaagtccaagtttgctg	atcgtgatgctgagaagtct

### Proliferation and apoptosis assays

Proliferating Müller cells and microglia were marked by the incorporation of bromodeoxyuridine (BrdU). BrdU was added to the culture medium for 2 hours (final concentration 10 μM), after which the cells were fixed in 4% paraformaldehyde for 60 minutes, washed with PBS, and incubated in 2N HCL in 1 × PBS for 60 minutes. After rinsing in 1 × PBS, cells were labeled with an anti-BrdU antibody overnight (G3G4, 1:200, Developmental Studies Hybridoma Bank, Iowa City, IA), followed by a goat anti-mouse antibody conjugated with Cy3 (Invitrogen, Carlsbad, CA). Cells were coverslipped in Vectashield mounting medium containing DAPI and imaged by epifluorescence microscopy. Images of 5 randomly chosen high-power fields were obtained for each well; the numbers of BrdU+ and DAPI+ cells in each field were counted and the percentage of the BrdU+ of the total cells present were calculated and averaged.

Cellular apoptosis in cultures was assessed by terminal deoxynucleotidyl transferase dUTP nick end labeling (TUNEL) using an assay kit according to the manufacture's instruction (Roche, Nutley, NH). A similar mounting and imaging procedure was performed as for BrdU labeling and the percentages of TUNEL-positive cells among total cells present were calculated and averaged.

### Photoreceptor neuroprotection assay

661W cells (a gift from Drs Zhongshu Tang and Xuri Li, National Eye Institute, Bethesda, MD), a photoreceptor-derived cell line, were plated in 96-well plates at a density of 1 × 10^4 ^cells/well. Hydrogen peroxide (H_2_O_2_, 0.1 mM) was added to induce oxidative stress-mediated photoreceptor cell death. The neuroprotective effects of Müller cell-conditioned media, as well as exogenous GDNF (AbCam, Cambridge, MA) and LIF (Prospec, East Brunswick, NJ) were assessed by adding them to H_2_O_2 _-stressed 661W cell cultures. Photoreceptor cell survival following 24-hour incubation was assessed using two separate methods according to the manufacturers' protocol: (1) a colorimetric microplate, tetrazolium salt-based, cell survival assay (Cell Counting Kit-8 Assay, Dojindo Molecular Technologies, Rockville, MD), and (2) a fluorescence-based, differential staining technique of live versus dead cells (LIVE/DEAD^® ^Fixable Dead Cell Stain Kit, Invitrogen, Carlsbad, CA). The percentageof survival cells were quantified and expressed as a percentage of the untreated control (not subject to H_2_O_2 _-mediated stress).

### ELISA

The protein levels of GDNF, LIF and CCL3 were measured using the following ELISA kits respectively according to their manufactures' instructions: 1) GDNF (Promega, Madison, WI), 2) LIF (R&D System, Minneaplos, MN), and 3) CCL3 (AdCam, ab100726, Cambridge, MA). Levels of IL-1β, IL6, TGF-β, ICAM and VCAM were measured with a commercial chemiluminescence-based ELISA assay (Searchlight, Aushon Biosystems, Billerica, MA).

### Nitrite concentration assay

NO was measured indirectly via its stable metabolite nitrite using the Griess reagent system (Promega, Madison, WI). The conditioned media was first incubated with sulfanilamide solution for 10 minutes at room temperature. N-naphthylethylenediamine was added to the mixture and incubated for 10 minutes. The absorbance was read in a plate reader at 550 nM and nitrite concentration was calculated by comparison to a standard reference curve.

### Evaluation of the effects of Müller cell-conditioned media on microglia

Fresh cultured microglia were plated in 6-well plates at the density of 1 × 10^6^/well. After 24 hours, the cells were washed 3 times with DMEM and incubated in the conditioned media collected from Müller cells following co-culture for another 24 hours. Microglia were harvested for mRNA analysis or fixed in 4% paraformaldehyde in PBS for 60 minutes for a proliferation assay.

### Microglial-Müller cell adhesion assay

The adhesion of microglia to Müller cells were assessed using an *in vitro *cell adhesion assay according to the manufacturer's instructions (Vybrant Cell Adhesion Assay Kit, Invitrogen, Carlsbad, CA). Müller cells were cultured similarly as before as: (1) alone, (2) co-cultured with unactivated microglia, or (3) co-cultured with activated microglia in 96-well transwell plates (Corning, Corning, NJ) with a seeding density of 5-8 × 10^3 ^cells/well in the lower chamber and microglia at a density of 0.5-1 × 10^4^cells/well in the upper chamber for 48 hours. The upper chamber was then removed leaving behind the post- cocultured Müller cells in the bottom chamber. Separately, fresh unactivated cultured microglia were collected, washed with PBS, resuspended in DMEM containing 5 μM Calcein-AM at the density 1-2 × 10^6 ^cells/ml for 30-60 minutes to allow for cell labeling. The suspension of labeled microglia was centrifuged, washed with PBS, and then resuspended at the density of 2 × 10^5 ^cells/ml in DMEM. A volume of 150 μl of the Calcein-AM-labeled cell suspension was added to the previously co-cultured Müller cells in the well-bottoms and incubated at 37°C for 90 minutes to allow for microglia-to- Müller cell adhesion. The wells were carefully washed 4 times with DMEM to remove non-adherent cells and 200 μl of PBS was added. Fluorescence in each well was measured using Spectramax MT (Molecular Devices Inc., Sunnyvale, CA) and expressed as a fraction of the fluorescence of the total cells added to each well.

### Microglial chemotaxis assay

Microglial chemotaxis was evaluated using 96-well transwell plates (Corning Incorporated, Corning, NY). The bottoms of the upper well-inserts contained polyester filters (8- μm pore size) that allowed for migration of cells from the upper chamber/well into the bottom chamber. Cultured microglia were harvested from cultures using 0.25% Trypsin-EDTA-mediated dissociation, centrifuged and washed with DMEM 3 times, resuspended in a 75 μl-volume of DMEM with 5% HI-FBS at a concentration of 1-2 × 10^5 ^cells/ml, and then seeded into the upper well insert. Conditioned media (125 μl) from previously co-cultured Müller cells were transferred into the bottom chamber. Additional bottom chambers also contained (1) non-conditioned medium (DMEM without serum) as a negative control, and (2) CCL2 (100 ng/ml in PBS) as a positive control. After incubation for 2 hrs at 37°C to allow for microglial migration, the top side of the insert filter was wiped carefully with a Q-tip to remove superficially adherent cells and the filter was removed. The filter was fixed in 4% paraformaldehyde, washed with PBS, stained with DAPI to mark the cells in the filter, and then mounted on a slide. Labeled cells that have migrated to the bottom side of the filter were imaged and counted.

### Intravitreous injections in experimental animals

Adult C57BL/6 mice were anesthetized with an intraperitoneal injection of ketamine (90 mg/kg) and xylazine (8 mg/kg). A small penetrating incision into the vitreous cavity was made in the eye directly behind the limbus using a 30 gauge needle under a dissecting microscope (Olympus, model SZX16; Central Valley, PA). A 1 μl volume of 1 × PBS containing different concentrations of lipopolysaccharide (LPS) was injected the vitreous using a Hamilton microliter syringe (Hamilton Company, model 701, Reno, NV). Animals were euthanized 3 days after intravitreal injection and ocular tissues obtained for analysis.

### Statistical analysis

Statistical analyses were performed using statistical software (Graphpad, San Diego, CA, USA). Comparisons between 3 or more data groups were performed using one-way analyses of variance (ANOVA), and comparisons between pairs of group means performed with the Tukey-Kramer multiple comparison test. A P value < 0.05 was set as the basis for rejecting the null hypothesis (i.e. that the group means for data groups being compared do not differ significantly from each other). In all graphical representations, the error bars indicate standard error (SE).

## Results

### Effect of microglial co-culture on morphological features of cultured Müller cells

**Figure 1:**
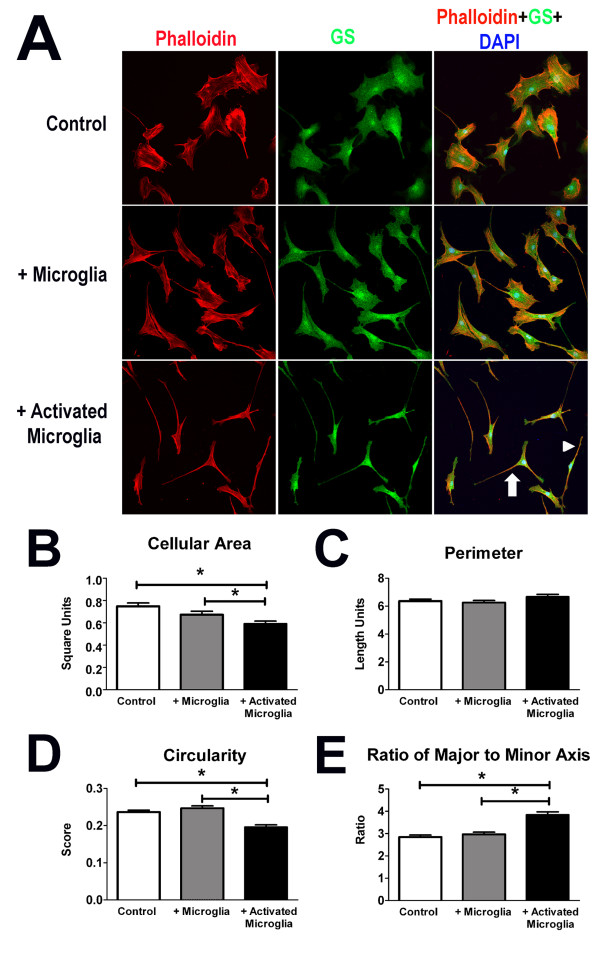
**Microglia-coculture induces alterations in cellular morphology in cultured Müller cells**. **(A) **Morphological features of cultured mouse Müller cells were visualized by immunolabelling of the actin cytoskeleton (with phalloidin, *red*), cytoplasm (with glutamine synthetase (GS), *green*), and nuclei (with DAPI, *blue*). Representative examples of Müller cells cultured alone (*top*), co-cultured with mouse microglia (*middle*), and cultured with microglia previously activated with lipopolysaccharide (LPS, 1 μg/ml) (*bottom*), are shown. Müller cells cultured alone were similar to those co-cultured with unactivated microglia in having broad lamellipodia and a symmetrical cell shape (*top and middle rows*) while co-cultured with activated microglia (*bottom row*) had elongated spindle (*arrowhead) *or multipolar (*arrow*) morphologies. Quantitative shape analyses in terms of morphological parameters of cellular area **(B)**, perimeter **(C)**, circularity **(D) **and elongation factor **(E) **revealed significant decreases in cellular area and increases in cellular elongation, without changes in overall cellular perimeter (n = 348 to 363 cells per group, * indicates p values < 0.05 for comparisons, one-way ANOVA).

We investigated the nature of direct retinal microglia-Müller cells interactions using an *in vitro *co-culture system. Mouse Müller cells were either (1) cultured alone without retinal microglia, (2) co-cultured with cultured mouse retinal microglia, or (3) co-cultured with microglia that had been previously activated by LPS (1 μg/ml). After 48 hours of co-culture, Müller cells that were cultured alone or with non-activated microglia retained a symmetrical, flattened cell shape with broad lamellipodia, while those co-cultured with activated microglia underwent a morphological transition into an elongated spindle-shaped or multipolar morphology with the loss of lamellipodial structures (Figure 1A). Quantitative morphological analysis demonstrated that this transition involved a reduction in cell area (Figure 1B), no change in cell perimeter (Figure 1C), a reduction in circularity (Figure 1D), and an increased elongation index (Figure 1E).

### Effect of microglial co-culture on Müller cell gliosis, proliferation, and apoptosis

**Figure 2:**
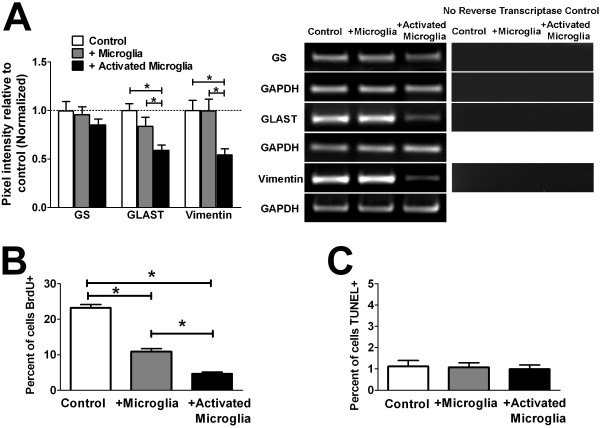
**Influence of microglia on Müller cell gliosis, proliferation, and apoptosis**. (**A**) The influence of microglia on Müller cell gliosis was assessed by evaluating mRNA expression of genes typically altered in gliosis by semi-quantitative RT-PCR: glutamine synthethase (GS), glutamate aspartate transporter (GLAST), and the intermediate filament, vimentin. Representative gel images for the PCR amplification of each mRNA species, with parallel controls from which reverse transcriptase is omitted from the amplification reaction (to confirm the absence of genomic DNA amplification), are shown (*right*). While the expression levels of GS in Müller cells were not statistically distinct between the different co-culture conditions, levels GLAST and vimentin, typically elevated in Müller cell gliosis, were significantly decreased following co-culture with activated microglia. These results demonstrate that changes induced by microglia co-culture differed from those associated with typical Müller cells gliosis. (**B**) Proliferating Müller cells in culture were marked by the incorporation of BrdU and the number of proliferating cells counted and expressed as a percentage of cells present. Co-culture with unactivated microglia induced a significant decrease in Müller cell proliferation, which was further decreased with co-culture with activated microglia. (**C**) Müller cells undergoing apoptosis in culture were marked with TUNEL-labeling. The percentage of apoptotic Müller cells was low and similar between all three co-culture conditions. (* indicates p < 0.05 for comparisons, one-way ANOVA with Tukey-Kramer multiple comparison test, n = 6-8 replicates from two independent experiments).

To investigate if microglia co-culture induced changes typical of Müller cell gliosis, genes whose expression levels were associated with gliotic changes were assessed in Müller cells following co-culture. While the mRNA levels of glutamine synthetase (GS) for all 3 co-culture conditions were relatively stable, co-culture with activated microglia did not increase levels of GLAST and vimentin as are typically associated with gliotic changes, but instead induced significant decreases in the levels of both genes (Figure 2A). Another intermediate filament protein which is typically increased in gliosis, GFAP, was expressed by Müller cells at very low levels for all 3 co-culture conditions (data not shown). Changes in Müller cell mRNA gene expression following co-culture with activated microglia were likely due to microglial factors rather than contaminating traces of LPS used in the activation of microglia; additional controls incorporating empty inserts exposed to LPS and then washed produced similar results as controls incorporating empty inserts alone (data not shown).

Müller cell proliferation, as measured by BrdU incorporation, was interestingly decreased as a result of co-culture with both unactivated and activated microglia (Figure 2B). Taken together, characteristics of Müller cell gliosis such as hypertrophy, increased mRNA expression of intermediate filament proteins, and cellular proliferation, were not upregulated following microglial co-culture, but were conversely downregulated. Microglial co-culture on the other hand did not induce differences in levels of cellular apoptosis, as assessed by TUNEL labeling, which were at low levels for all co-culture conditions (Figure 2C).

### Increased neuroprotective effects of Müller cells following co-culture with activated microglia

**Figure 3:**
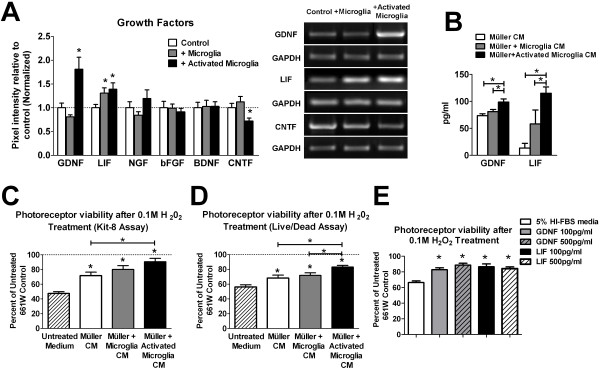
**Müller cell expression of growth factors and neuroprotective function following microglial co-culture**. (**A**) Semi-quantitative RT-PCR comparing mRNA levels of growth factors in Müller cells following microglial co-culture. Expression of growth factors GDNF and LIF were significantly elevated in Müller cells co-cultured with activated microglia. Expression levels of NGF, bFGF, and BDNF were similar between the three groups, while CNTF levels were slightly reduced. Representative gel images for genes whose expression were significantly changed following co-culture are shown (*right*). (**B**) ELISA quantification of protein levels of GDNF and LIF in the conditioned media of Müller cells following microglial co-culture. Relatively elevated levels of these growth factors were found in conditioned media from Müller cells co-cultured with activated microglia. (**C, D**) Neuroprotective function of Müller cells following microglial co-culture were evaluated by assessing the ability of conditioned media from co-cultured Müller cells to rescue photoreceptor cells (661W cells) from H_2_0_2_-induced oxidative cell death. Following Müller cell-microglia co-culture, microglia-containing cell inserts were removed, fresh medium was added to the resulting Müller cell cultures and left to condition for 24 hours. These conditioned media were added to 661W cells in the presence of 0.1 M H_2_0_2_. 661W cells exposed to 0.1 M H_2_0_2 _in regular unconditioned media served as controls. Cell viability of H_2_0_2_-exposed 661W cells were evaluated with a tetrazolium-based cell counting Kit-8 assay (in C) and a differential cell-staining (Live/Dead) assay (in D). Müller cell-media from all co-culture conditions exerted significant neuroprotective effect relative to unconditioned medium (marked by * over individual bars) but that from Müller cells co-cultured with activated microglia exerted a greater neuroprotective effect relative to Müller cells cultured without microglia. (E) Neuroprotective effect of exogenous GDNF and LIF on photoreceptor cells undergoing oxidative stress. GDNF and LIF (100 pg/ml and 500 pg/ml) were added to 661W cells in the presence of 0.1 M H_2_0_2_. 661W cells exposed to 0.1 M H_2_0_2 _in regular unconditioned media served as a control. Additions of GDNF and LIF were able to significantly increase 661W cell survival as evaluated with a cell counting Kit-8 assay. No significant dose-dependent effect was observed in the range of concentrations used. (* indicates p < 0.05 for comparisons, one-way ANOVA with Tukey-Kramer multiple comparison test, n = 6-8 replicates from two independent experiments).

Previous studies have demonstrated that Müller cells are able to express and secrete trophic factors that can provide neuroprotection to retinal neurons and photoreceptors [8,20-22]. We examined the effects of microglial co-culture on the mRNA expression of trophic factors in Müller cells. We observed that Müller cells exposed to activated microglia expressed higher mRNA levels of glial-derived neurotrophic factor (GDNF) and leukemia inhibitory factor (LIF), compared to Müller cells that were cultured alone. Nerve growth factor (NGF), basic fibroblast growth factor (bFGF) and brain-derived growth factor (BDNF) mRNA expression levels were not markedly changed, while that of ciliary neurotrophic factor (CNTF) was slightly decreased (Figure 3A). Measurements of protein levels of GDNF and LIF in the conditioned media using ELISA also demonstrated increased production of these factors by Müller cells that had been exposed to activated microglia (Figure 3B). We investigated if these expression changes in Müller cell growth factors induced by microglia-derived signals can confer differences in the neuroprotective capability of Müller cells. Following microglial co-culture, Müller cells were incubated with fresh media for another 24 hours, and the resulting conditioned media collected. We employed an *in vitro *neuroprotection assay in which conditioned Müller cell media from the 3 different culture conditions were evaluated for their ability to rescue photoreceptor cells (661W cell line) from oxidative stress-induced cell death. We found that while Müller cell-conditioned media from all 3 co-culture conditions were able to increase photoreceptor survival, conditioned media from Müller cells exposed to activated microglia induced a significantly higher photoreceptor survival rate compared with the media from Müller cells cultured alone (Figure 3C-D). These significant increases in photoreceptor survival rates can also be induced by the exogenous addition of GDNF or LIF (100-500 pg/ml) to the culture media (Figure 3E). Taken together, these results indicate that signals from activated microglia are capable of amplifying the neuroprotective effects of Müller cells, likely by the increased protein expression of trophic factors, including GDNF and LIF.

### Upregulation of inflammatory cytokines in Müller cells following co-culture with activated microglia

**Figure 4:**
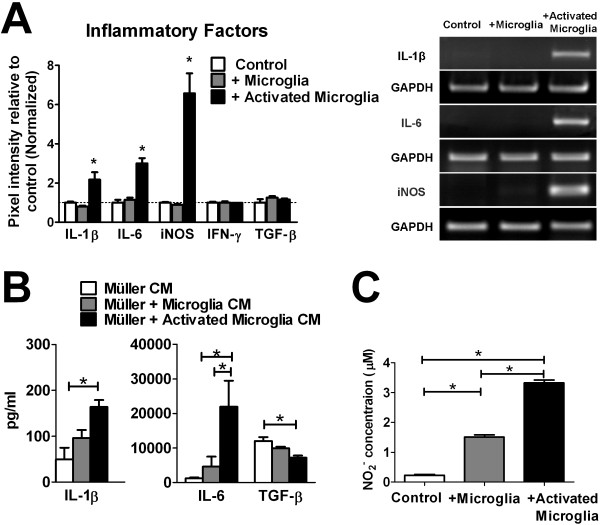
**Influence of microglia on Müller cell expression of inflammatory factors**. (**A**) Semi-quantitative RT-PCR comparing mRNA levels of inflammatory factors in Müller cells cultured alone (*white bars*), or co-cultured with unactivated (*gray bars*) or LPS-activated (*black bars*) microglia. mRNA levels of inflammatory cytokines IL-1β and IL-6, as well as iNOS, were significantly elevated in Müller cell co-cultured with activated microglia, compared with those cultured alone or cultured with unactivated microglia. Expression levels of IFN-γ and TGF-β were similar between the three groups. Representative gel images for genes whose expression were significantly changed following co-culture are shown (*right*). (**B**) Protein levels of cytokines in the conditioned media of Müller cells 24 hours following co-culture were measured with ELISA. Inflammatory cytokines, IL-1β and IL-6, were both elevated, while levels of TGF-β were slightly decreased. (**C**) Nitrite concentrations in conditioned media following co-culture were elevated in Müller cells co-cultured with activated microglia, consistent with the increased expression for iNOS in Müller cells. (* indicates p < 0.05 for comparisons, one-way ANOVA with Tukey-Kramer multiple comparison test, n = 6-8 replicates from two independent experiments).

As Müller cells can respond to injury and disease by the production of inflammatory mediators [23-25], we investigated how microglial co-culture can influence on Müller cell expression of inflammatory cytokines and enzymes. We observed that Müller cells exposed to activated microglia expressed higher mRNA levels of interleukin-1 beta (IL-1β), Interleukin-6 (IL-6), and inducible nitric oxide synthases (iNOS), relative to Müller cells exposed to non-activated microglia or to unexposed control Müller cells (Figure 4A). mRNA expression levels of TNFα were very low for all co-culture conditions and could not be accurately quantified (data not shown). mRNA levels of interferon-gamma (IFN-γ) and transforming growth factor beta **(**TGF-β) were similar in all culture conditions. ELISA analysis of conditioned media also revealed that Müller cells exposed to activated microglia secreted significantly higher levels IL-1β and IL-6, and lower levels of TGF-β (Figure 4B). Measurement of nitrite levels in conditioned media also demonstrated increased production in Müller cells co-cultured with both non-activated and activated microglia, consistent with the increased levels of iNOS mRNA expression in those co-cultures (Figure 4C).

### Müller cell regulation of microglial activation following previous microglial co-culture

**Figure 5:**
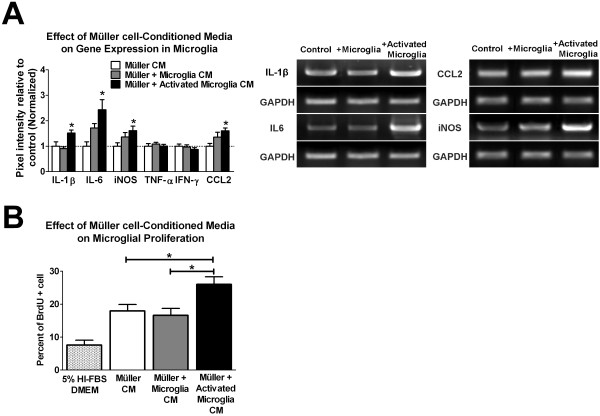
**Müller cells, following co-culture with microglia, can induce reciprocal activation of retinal microglia**. Following microglial co-culture, fresh media were added to Müller cells from each co-culture condition, left to condition for 24 hours, and then collected and tested for the ability to induce microglial activation. These conditioned media were added to fresh, unactivated cultured microglia for 24 hours. The ability of conditioned media to induce reciprocal microglial activation was assessed by measuring microglial mRNA expression of proinflammatory factors and microglial proliferation. (**A**) Conditioned media from Müller cells co-cultured with activated microglia were able to induce the highest levels of IL-1β, IL-6, iNOS, and CCL2 expression in microglia. Microglial expression levels of TNF-α and IFN-γ remained unchanged between three groups. Representative gel images for genes whose expression were significantly changed following co-culture are shown (*right*). (**B**) Microglia proliferation, as measured by BrdU incorporation, was significantly elevated in the conditioned media from Müller cells exposed to activated microglia, relative to other conditioned media. (* indicates p < 0.05 for comparisons, one-way ANOVA with Tukey-Kramer multiple comparison test, n = 6-8 replicates from two independent experiments).

We reasoned that while microglia can signal to Müller cells to alter their expression and secretion of multiple factors, these alterations in Müller cells may consequently induce reciprocal signaling back to microglia. These bidirectional feedback signals between microglia and Müller cells may in fact constitute a coordinated response following the induction of injury or disease. As activated microglia induced in Müller cells the production and secretion of inflammatory factors, we hypothesized that these altered Müller cells may also in turn influence microglial physiology and activation. Following microglial co-culture, Müller cells were incubated with fresh media for another 24 hours, and this resulting conditioned media was added to new, unactivated microglia. These microglia were then harvested after 24 hours to assay their mRNA expression. We found that the conditioned media from Müller cell-activated microglia co-cultures were able to induce in fresh microglia significant increases in IL-1β, IL-6, iNOS, and chemokine (C-C motif) ligand 2 (CCL2) (Figure 5A). The same conditioned medium was also able to induce the highest increases in microglial proliferation as measured by BrdU incorporation (Figure 5B). To ensure that these microglial changes in response to Müller cell derived factors rather than from contaminating LPS, we performed experiments with an additional control empty insert that was also treated with LPS; these control experiments demonstrated results similar to the original control (data not shown). Together, it appears that Müller cell changes induced by activated microglia can in turn induce the activation of fresh microglia as evidenced by increased microglial proliferation and inflammatory gene expression.

### Upregulation of Müller cell-microglia adhesion following Müller cell exposure to activated microglia

**Figure 6:**
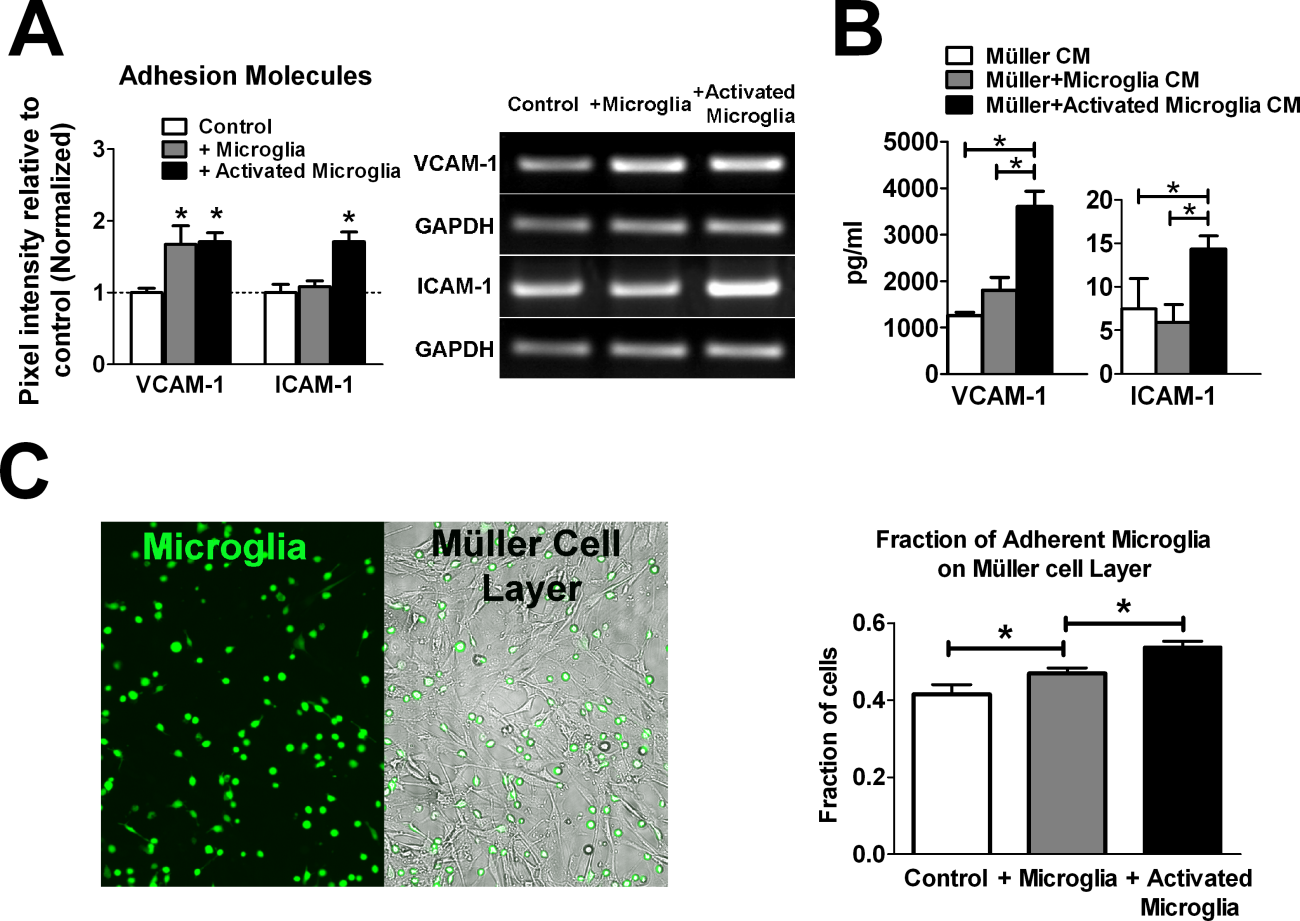
**Influence of microglia on Müller cell expression of adhesion molecules and adhesion properties**. **(A) **Semi-quantitative RT-PCR comparing mRNA levels of adhesion molecules, VCAM-1 and ICAM-1, in Müller cells cultured alone (control, *white bars*), co-cultured with unactivated (*gray bars*) or activated (*black bars*) microglia. The mRNA levels of VCAM-1 were significantly elevated in Müller cells co-cultured with unactivated and activated microglia, compared with Müller cells cultured alone. ICAM-1 was significantly elevated only in Müller cells that were co-cultured with activated microglia. Representative gel images for genes whose expression were significantly changed following co-culture are shown (*right*). **(B) **Protein levels of adhesion molecules in Müller cell-conditioned media were measured with ELISA. Relatively elevated levels of VCAM-1 and ICAM-1 were found in conditioned media from Müller cells co-cultured with activated microglia. **(C) **The ability of Müller cells following co-culture to function as an adhesive substrate for microglia cells was assessed by a cell-adhesion assay. Unactivated microglia, labeled vitally with Calcein-AM (*left panel*, in green), were seeded onto Müller cells following 48-hr co-culture and allowed to adhere (*right panel*, Müller cells seen in bright-field). Non-adherent microglia were washed off and the remaining cells were counted and expressed as a fraction of total microglial cells added. Müller cells that had previously been co-cultured with activated microglia were found to induce the highest levels of adhesion, followed by Müller cells that had previously been co-cultured with unactivated microglia. (* indicates p < 0.05 for comparisons, one-way ANOVA with Tukey-Kramer multiple comparison test, n = 6-8 replicates from two independent experiments).

Müller cells possess a radially-oriented cellular geometry with cellular processes that traverse the entire thickness of the retina. On the other hand, retinal microglia, under normal resting conditions, possess a predominantly horizontal cellular orientation and are located almost exclusively in the inner retinal layers up to the outer plexiform layer. However, under conditions of injury, disease, and aging, activated microglia are able to assume a vertical cellular orientation and migrate in a radial direction across retinal lamina [26-29]. How microglia and Müller cells interact with each other in the context of these changes is incompletely understood. In these translocations, microglia may interact with radial Müller cell processes via adhesive cellular contacts as a physical scaffold for attachment and cellular movement. In assays for the expression of adhesion molecules, we found that following co-culture with activated microglia, Müller cells demonstrated higher mRNA and protein expression levels of vascular cell adhesion molecule-1 (VCAM-1) and intercellular adhesion molecules (ICAM-1) compared to Müller cells cultured alone (Figure 6A, B). This increased expression of adhesion molecules in Müller cells co-cultured with activated microglia suggest that they may present a more adherent substrate for microglia attachment. To assess microglial adhesion to Müller cells, we employed a cell-adhesion assay in which fresh cultured microglia were pre-labeled with Calcein-AM and then seeded on Müller cells surfaces following co-culture. Non-adhering microglia were removed by a standardized washing regimen, and the retained adherent cells counted. We found that Müller cells previously co-cultured with activated microglia were more adherent than those co-cultured with non-activated microglia or cultured alone (Figure 6C, D). These results indicate that prior exposure to activated microglia had modified Müller cell expression of surface molecules in a way that promoted microglia-Müller cell adhesion.

### Upregulation of microglia chemotaxis by Müller cells following exposure to activated microglia

**Figure 7:**
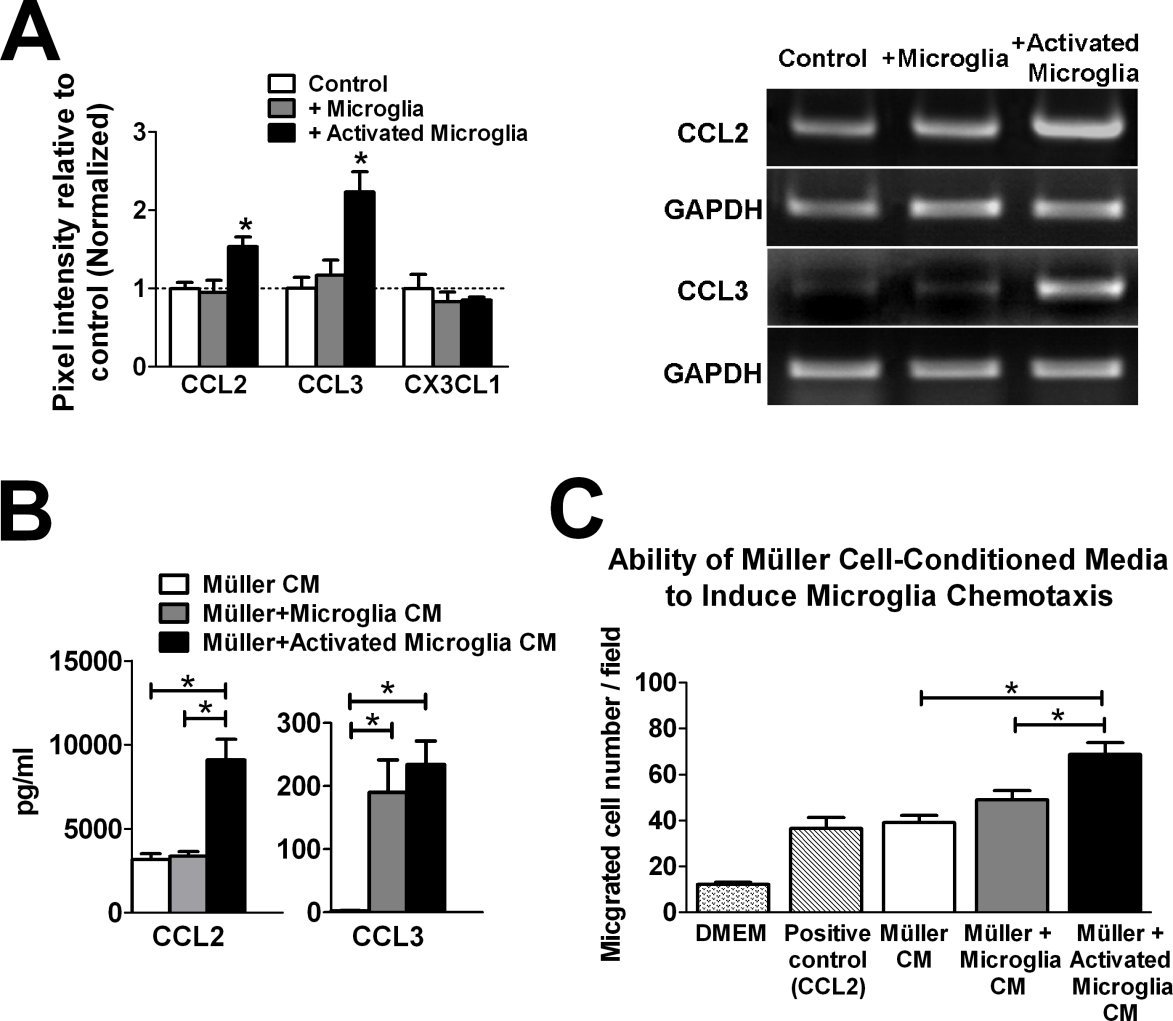
**Müller cell expression of chemotactic cytokines following microglia co-culture**. **(A) **Quantitative RT-PCR comparing mRNA levels of chemotactic cytokines in Müller cells cultured alone (*control, white bars*), or co-cultured with unactivated (*gray bars*) or activated microglia (*black bars*). The mRNA levels of CCL2 and CCL3 were significantly elevated in Müller cell co-cultured with activated microglia, compared with those cultured alone or cultured with unactivated microglia. Expression levels of CX3CL1 were unchanged between the three groups. Representative gel images for genes whose expression were significantly changed following co-culture are shown (*right*). **(B) **Protein levels of chemokines CCL2 and CCL3 in Müller cell-conditioned media were measured with ELISA. Relatively elevated levels of these chemokines were found in conditioned media from Müller cells co-cultured with activated microglia. **(C) **The ability of Müller cell-conditioned media to induce the chemotaxis of microglia was assayed using a modified Boydon chamber. Conditioned media from Müller cells exposed to activated microglia exerted the largest chemoattractive effect on microglia (*black bar*), followed by media from Müller cells co-cultured with unactivated microglia (*gray bar*), relative to media from Müller cells that were cultured alone (*white bar*). CCL2 (100 nM in DMEM medium) was used as a positive control for microglial chemotaxis. (* indicates p < 0.05 for all comparisons, one-way ANOVA with Tukey-Kramer multiple comparison test, n = 6-8 replicates from two independent experiments.)).

In addition to serving as a physical substrate for adhesion-based translocation, Müller cells may be induced by microglia to secrete chemotactic cytokines that can guide microglial migration. To address this, we assayed the expression of cytokines that have been previously demonstrated to induce chemotaxis in Müller cells. We found that following co-culture, the mRNA levels of CCL2 and CCL3 were significantly elevated in Müller cells which had been co-cultured with activated microglia. mRNA levels of chemokine (C-X3-C motif) ligand 1 (CX3CL1) was however unchanged (Figure 7A). Relatively higher protein levels of CCL2 and CCL3 were similarly found in the conditioned media of these Müller cells by ELISA (Figure 7B). Conditioned Müller cell media were also assayed for their ability to induce microglia migration using a modified Boyden chamber. We found that while conditioned media from Müller cells cultured alone or from co-cultured with unactivated microglia induced microglial migration at levels similar to that induced by exogenous CCL2 (positive control), Müller cells co-cultured with activated microglia demonstrated a significantly greater induction of microglial migration (Figure 7C). Taken together, this indicated that Müller cells, in response to inductive signals from activated microglia, increased their ability to facilitate microglial migration, likely as a result of increases in chemokine expression.

### Müller cell-microglia interactions following microglial activation *in vivo*

**Figure 8:**
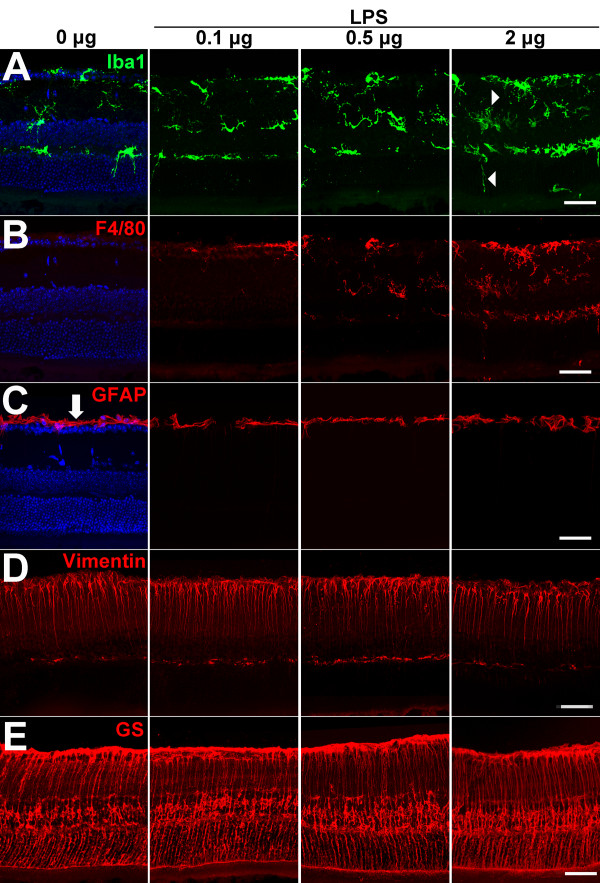
***In vivo *retinal microglia and Müller cell responses to retinal microglial activation induced by intravitreal injection of lipopolysaccharide (LPS)**. *In vivo *activation of retinal microglia was induced by intravitreal injection of different total amounts of LPS (0 μg, 0.1 μg, 0.5 μg, 2 μg) dissolved in 1 × PBS. Eyes injected with only 1 × PBS (0 μg LPS) served as controls (*left column*). Animals were sacrificed and enucleated 3 days after intravitreal injection and cryosections prepared from the globes. (**A**) Iba1 immunolabeling (*green*) of retinal sections show that following LPS injection, microglia exhibit a dose-dependent increase in cell density and Iba-1 immunopositivity, as well as an increase in the number of vertically oriented processes (*arrowheads*) as compared to PBS-injected controls. (**B**) F4/80 immunolabeling (*red*), a marker of microglial activation, demonstrates a dose-dependent increase in the density of immunoreactive microglial cells following LPS injection. (**C**) GFAP immunolabeling (*red*) located only in astrocytic processes (*arrow*) in the PBS-injected control, did not change in its localization following LPS injection, indicating that typical Müller cell gliosis, exemplified by increased GFAP expression, did not occur under these conditions. Vimentin (**D**) and glutamine synthetase (GS) (**E**) immunolabeling (*red*), located in Müller cell process, was present under all conditions and were not noticeably different in intensity. No marked changes in the morphology of Müller cells were noted. Scale bar = 50 μM.

In order to assess if features of *in vitro *interactions between microglia and Müller cells were recapitulated *in vivo*, we activated retinal microglia in the mouse retina by intravitreal injections of LPS at three increasing doses of 0.1 μg, 0.5 μg, and 2 μg. Three days following injection, experimental animals were sacrificed and their retinas sectioned for immunohistochemical analysis. LPS injection induced microglial activation in a dose-dependent manner as evidenced by the increasing intensity of immunohistochemical staining of microglia by Iba-1 (Figure 8A) and F4/80 (Figure 8B). With activation, Iba-1-labeled microglia were also observed to extend a greater number of processes in the radial direction and adopt an elongated morphology that traversed multiple retinal layers (Figure 8A, *right*). We also examined Müller cells at this time point for immunohistochemical and morphological evidence of typical gliotic changes. As in our *in vitro *experiments, Müller cells in the retina, which are mostly immunonegative for GFAP under resting and control conditions, failed to develop GFAP immunostaining following LPS injection over this acute time-frame (3 days) (Figure 8C), as previously described [30]. Immunostaining for vimentin, another intermediate filament protein, upregulated in typical Müller cell gliosis, did not change detectably following LPS administration (Figure 8D). The morphology of Müller cells, as revealed by staining with glutamine synthetase (GS), also remained relatively unchanged without evidence of process hypertrophy (Figure 8E). Taken together, our *in vivo *data corroborate *in vitro *observations that Müller cells in the acute aftermath of microglia activation do not exhibit changes described in typical gliosis.

**Figure 9:**
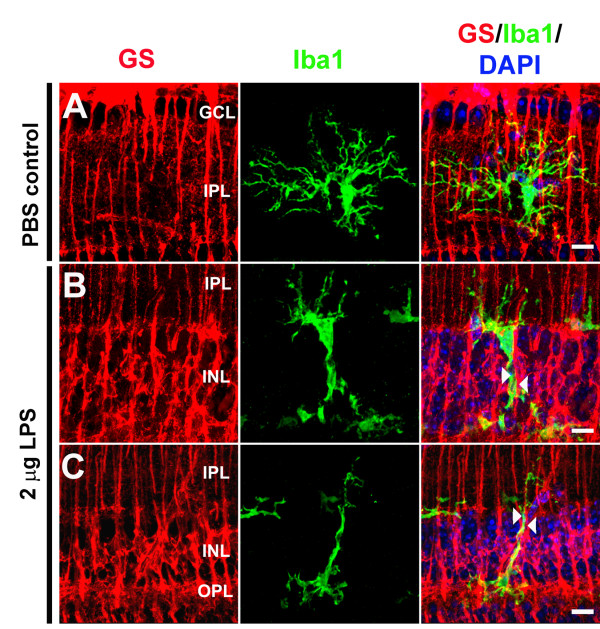
**Physical interaction of microglia-Müller cell processes following microglial activation *in vivo***. *In vivo *activation of retinal microglia was induced by intravitreal injection of lipopolysaccharide (LPS, 2 μg in 1 μl of 1 × PBS). Control eyes were injected with 1 μl of 1 × PBS alone. Retinal cryosections were prepared 3 days after injection and immunolabeled with glutamine synthetase (GS) (*red*) to mark Müller cell processes, Iba-1 (*green*) to mark microglia, and DAPI (*blue*) to mark retinal cell nuclei. **(A) **In PBS-injected control eyes, ramified processes of microglia in the inner retina are oriented predominantly in the horizontal plane of the retina and show minimal interaction or fasciculation with the vertically oriented, GS-positive, Müller cell processes. (**B, C**) In LPS-injected eyes, microglia in the inner retina demonstrate a more vertical orientation of their processes compared to controls. Microglial processes were observed to be juxtaposed in close physical association with parallel Müller cell processes. Close fasciculation between the vertical processes of both cell types can be observed (*arrowheads*), suggesting cellular adhesion and physical interaction between Müller cell-microglia processes. In examples in which vertically oriented microglia appear to be migrating in the radial direction, Müller cell processes appear to be acting as a adhesive scaffold that guide microglia orientation and translocation. Scale bar = 10 μM.

In order to examine closely the physical *in vivo *interaction between Müller cells and microglia in terms of their morphological and spatial relationships, we imaged retinal sections with confocal microscopy under high magnification. In PBS-injected control eyes, retinal microglia (labeled with Iba-1 antibody) did not show any overt morphological or immunohistochemical signs of activation. The horizontally-oriented, ramified processes of microglia interdigitated with the radially-oriented, GS-labeled Müller cell processes but the points of contact or fasciculation between the two cell types were few (Figure 9A) (Additional File 1). In eyes that were intravitreally injected with 2 μg of LPS, retinal microglia demonstrated a less-ramified and more radially-oriented morphology, with processes that tended to be distributed *across*, rather than within, retinal lamina. High-magnification confocal imaging of the retina revealed that radially-oriented microglial processes tended to fasciculate closely with Müller cell processes, which extended to most of the length of the microglial cell (Figure 9B, C). This close juxtaposition of microglial-Müller cell processes indicates an adhesive cell-cell interaction, suggesting that microglia may rely on Müller cell-based contacts as a scaffold to translocate through retinal tissue following activation (Additional File 2 and 3).

## Discussion

### Acute Müller cell responses to microglial activation do not resemble "typical" gliosis

In the current study, we have employed an *in vitro *co-culture model of cultured mouse retinal cells to examine the direct cellular interactions between microglia and Müller cells. While *in vitro *systems may be limited in how accurately they reflect *in situ *properties, they enable direct assessment of interactions between two cell types, the results of which can then be confirmed in *in vivo *systems [31]. Our use of a two-chambered co-culture system permits cellular interactions via soluble factors to occur, followed by the clean separation of the two populations of cells for analysis. In our study, we have also focused primarily on the acute, rather than the long-term changes, induced following microglial activation. We reasoned that as activation states of both microglia and Müller cells can vary significantly as a function of time following induction [32,33], the presence and nature of cellular interactions may be most directly assayed soon after the initiation of cellular communication.

While microglial and Müller cell responses have been detected and described in a wide variety of retinal injuries and diseases [6,7], direct communications between the two cell types in these contexts are less well understood. In general, while there is evidence that microglial activation precedes macroglial responses at the initiation of tissue injury [4,5], if and how activated microglia in the retina communicate to Müller cells to coordinate an overall injury response remains unclear. Our results show that cultured Müller cells are highly responsive to the presence and activation status of retinal microglia, and significantly alter their cell morphology, gene expression, and cellular interactions as a consequence. We observed that following acute exposure to activated microglia, Müller cells in culture transition from a flattened, lamellipodial shape to an elongated spindle-shaped morphology, demonstrate decreased proliferation, and downregulate their expression of vimentin and GLAST. These changes differ from some previously described signature features of typical Müller cell gliosis which include cellular hypertrophy, proliferation, and the upregulation of intermediate filaments (e.g. GFAP, vimentin) [8]. Our *in vivo *observations of Müller cells 3 days following intravitreal LPS injection similarly lacked these features of hypertrophy, GFAP/vimentin upregulation, and proliferation in Müller cells, despite prominent signs of concurrent microglial activation. These differences suggest that early responses of Müller cells to microglial activation in this case may differ significantly from the gliotic changes induced in other injury models. These differences may have arisen as a result of longer term consequences of sustained microglial activation or from secondary indirect influences from other retinal cell types. For example, in retinal detachment, outer retinal photoreceptor degeneration occurs rapidly, and may directly induce typical gliotic changes in Müller cell endfeet [34]. In a similar model involving intravitreal LPS, Liu et al. [30] have reported differences in acute (1-3 days) and long-term (7-14 days) gene expression changes in Müller cells following LPS administration; in these experiments GFAP immunopositivity was absent in the first week post-injection and emerged only after 7-14 days. Interestingly, short-term responses of brain astrocytes to activated microglia also involve an absence of cellular hypertrophy and a downregulation of both GFAP and vimentin [35]. These differences between the acute Müller cellresponses to activated microglia versus "typical" gliosis may also help discern different modes of gliotic reactions occurring in differing cellular and temporal contexts and entail distinct functional significances.

### Neuroprotective consequences of Müller cell responses to microglial activation

Our results show that Müller cells increase their expression of growth factors, GDNF and LIF in response to microglial activation. The functional significance of this response was indicated by the ability of conditioned media from Müller cells exposed to activated microglia to provide increased levels of neuroprotection to photoreceptors undergoing oxidative stress injury. These results suggest that retinal microglia following injury/activation can direct Müller cells to provide neuroprotective signaling onto photoreceptors. Previous reports have documented the ability of GDNF [36,37] and LIF [22,38] to confer photoreceptor neuroprotection; photoreceptors have also been shown to express receptors for GDNF (GFRα1 and 2) [21,39], and to respond directly to LIF by STAT3 activation [40]. We observed that the upregulation of growth factors by Müller cells also occurred concurrently with the downregulation of intermediate filament proteins, suggesting that these two cellular responses may bear a relation to each other. Consistent with this notion, transgenic mice deficient in GFAP and vimentin in the aftermath of retinal detachment exhibit less photoreceptor degeneration and glial scar formation compared to wild type controls [41]. Measures that decrease Müller cell GFAP/vimentin expression, either by pharmacological inhibition [42] or using lentiviral-mediated gene knockdown methods [43], have also resulted in increased neuronal survival rates. While the mechanisms for how changes in intermediate filament expression regulate neuroprotective functions are not known [44], our results indicate that adaptive Müller cell responses to early microglial activation, in inducing decreases in intermediate filament expression, may be part of an overall response with neuroprotective consequences for photoreceptors.

### Bidirectional inflammatory signaling between Müller cells and retinal microglia

Our results indicate that Müller cells respond to microglial activation by an upregulation of infllammatory mediators IL-1β, IL-6, and iNOS. These effects are likely mediated in response to inflammatory mediators from activated microglia such as IFN-γ, IL-1β, and TNF-α as described in previous reports [45-48]. This production of inflammatory mediators in response to signals from activated microglia suggests that Müller cells may be able to amplify inflammatory responses in the retina in a positive feedback loop. Indeed, we found that conditioned media from Müller cells previously co-cultured with activated microglia were able to induce significantly larger increases in inflammatory mediator mRNA expression (IL-1β, IL-6, iNOS, and CCL2) in fresh, non-activated microglia, compared to conditioned media from Müller cells cultured alone. This potential bidirectional feedback loop between microglia and Müller cells may allow the few microglia initially activated near the vicinity of a local injury or injection to extend the spatial locus of inflammatory response. Responding Müller cells in the vicinity may be able to transmit inflammatory signals across multiple retinal lamina as a function of their radial processes that extend across the thickness of the retina, and also tangentially via lateral communications between adjacent Müller cells, possibly involving calcium waves [49]. These Müller glia-microglia responses may underlie a mechanism in which an initial detection of injury in a particular locus by microglia may be augmented in magnitude and in spatial scale to a broader adaptive injury response involving both cell types.

### Coordination of microglial recruitment and migration by Müller cells

Our results show that Müller cell responses to activated microglia in addition involve the increased production of adhesive proteins (VCAM-1 and ICAM-1) and chemotactic cytokines (CCL2 and CCL3). Following co-culture with activated microglia, the cell surfaces of Müller glia were also altered, resulting in greater adhesion to microglia. Conditioned media from these Müller cells were also able to induce significantly higher levels of microglial chemotaxis. These alterations suggest that Müller cells respond to microglial activation by producing signals that can induce microglial migration and recruitment and by presenting an adhesive and radially-oriented scaffold to guide these microglial translocations. In the uninjured resting state, retinal microglia have morphologies that are predominantly horizontal, a distribution that is limited to the inner retina, and a relatively stable soma position, despite prominent dynamism in their cellular processes [13,50]. However, in response to photoreceptor degeneration [51,52], light injury [53,54], or aging changes [26], retinal microglia are observed to translocate in the radial direction towards injury areas, including the outer retina. We postulate that Müller cells may play a role in the guidance and facilitation of this migration by the presentation of soluble chemotactic guidance cues [55,56] as well as physical cell-cell interactions in the form of an adhesive scaffold [57]. Interestingly, radial migration of microglia adhering to and guided by Müller cell vertical processes was observed during retinal development [58]. Our *in vivo *observations of Müller cell-microglial interactions following microglial activation by LPS provide corroborating evidence for this function. In the absence of activation, microglia in the retina have processes that are generally oriented orthogonally with respect to the radially-oriented Müller cell processes, with few direct cellular contacts. Following activation, microglial cells become more radially oriented in their morphologies with processes that fasciculate closely with the radial processes of Müller cells. Taken together, our data support the notion that Müller cells respond to microglial activation with an increase in cell-cell contacts and chemokine secretion that facilitate and guide the radial migration of microglial cells in inflammatory responses in the retina.

One caveat in interpreting these *in vivo *observations is that intravitreal LPS may not only activate microglia which then influence Müller cells, but may also act on Müller cells directly. Cultured astrocytes have been found to produce a responses to LPS stimulation but to a markedly lower degree compared to microglial cells [59], to the extent that the few contaminating microglia in mixed astroglial cultures may overrepresent astrocyte responses to inflammatory stimuli [60]. As we did not observe significant changes in Müller cells in terms of morphology or immunohistochemical staining of gliosis markers at the point that microglial changes were already prominent, we similarly expect that the overall retinal effects to intravitreal LPS would be primarily driven by microglial rather than Müller cell responses, although the contribution of the latter cannot be completely ruled out.

In conclusion, our *in vitro *and *in vivo *observations indicate that the injury responses of activated Müller cells and microglia in the retina are not independent but involve bidirectional feedback signals that help initiate and propagate a coordinated adaptive response. The acute features of this program involve (1) an increased neuroprotection through upregulated expression of Müller cell-derived growth factors, (2) a potentiation of microglia and Müller cell activation by proinflammatory positive feedback signaling, and (3) a facilitation of microglial migration within the retina as mediated by chemotactic signals and an adhesive Müller cell scaffold. These adaptive responses may represent mechanisms in the retina invoked by injury or disease that help to limit cell death while directing and amplifying inflammatory processes to restore homeostasis. Elucidating the nature of relationships between these cell types prominent in retinal pathology can potentially illuminate injury and disease mechanisms and highlight therapeutic strategies aimed at manipulating these responses.

## List of abbreviations used

BDNF: brain-derived neurotrophic factor; CNTF: ciliary neurotrophic factor; CCL2: chemokine (C-C motif) ligand 2; CCL3: chemokine (C-C motif) ligand 3; CX3CL1: chemokine (C-X3-C motif) ligand 1; ICAM: intercellular adhesion molecules; IFN-γ: interferon-gamma; IL-1β: interleukin-1 beta; IL-6: interleukin-6; iNOS: nitric oxide synthases; GDNF: glial cell-derived neurotrophic factor; GFAP: glial fibrillary acidic protein; GLAST: glutamate aspartate transporter; GS: glutamine synthetase; LIF: leukemia inhibitory factor; TGF-β: transforming growth factor beta; TNF-α: tumor necrosis factor alpha; VCAM: vascular cell adhesion molecule.

## Competing interests

The authors declare that they have no competing interests.

## Authors' contributions

MW was involved in study design, performed the experiments, analyzed the data, images, and statistics, and wrote the manuscript. WM contributed to cell-culture experiments and helped write the paper. LZ performed the *in vivo *experiments and helped write the manuscript. RNF contributed to imaging experiments and data analysis, and helped write the manuscript. WW was involved in study design, provided research support, and helped write the manuscript. All authors have read and approved the final version of the manuscript.

## Supplementary Material

Additional file 1 **Spatial relationship between Müller cells and microglia 3 days after intravitreal injection of 1 × PBS (control)**. Movie showing a 3-dimensional rotational representation of a "resting" microglia cell and surrounding Müller cell processes (also shown in Figure 9A) following surface rendering image processing. A typical ramified microglia cell (labeled with Iba-1, *green*) is shown with horizontal processes that interdigitate, but does not fasciculate, with the radially-oriented Müller cell processes (labelled with glutamine synthetase, *red*). Click here for file

Additional file 2 **Spatial relationship between Müller cells and microglia 3 days after intravitreal injection of LPS (2 μg)**. Movie showing a 3-dimensional rotational representation of a microglia cell and surrounding Müller cell processes (also shown in Figure 9B) following surface rendering image processing. Following LPS, retinal microglia (*green*) have multiple vertically-oriented processes that demonstrate closely fasciculating cell-cell contact with Müller cells (*red*). Click here for file

Additional file 3 **Spatial relationship between Müller cells and microglia 3 days after intravitreal injection of LPS (2 μg)**. Movie showing a 3-dimensional rotational representation of a microglia cell and surrounding Müller cell processes (also shown in Figure 9C) following surface rendering image processing. Following LPS, retinal microglia (*green*) have multiple vertically-oriented processes that demonstrate closely fasciculating cell-cell contacts with Müller cells (*red*). Click here for file
